# Supplementary material for: Impact of State Stroke Systems of Care Laws on Stroke Outcomes
Source: Healthcare (Basel). 2023 Oct 28;11(21):2842. doi: 10.3390/healthcare11212842 (PMC10648022; doi:10.3390/healthcare11212842)

SUPPLEMENTAL MATERIALS

Table S1. Average difference between actual and predicted outcomes for three percentiles of the distribution of states by number of years of outcome data after first SSOC [a] policy was in effect

	Time span after first policy		
	Short-term<br>25th percentile	Mid-term<br>50th percentile	Long-term<br>75th percentile
Outcome 1: Proportion of certified PSCs [b]<br>Number of years (states) in the percentile	4<br>(n=22)	7<br>(n=12)	9<br>(n=7)
Mean difference between observed and actual outcome in percentage points [90% CI]	2.7<br>[0.4, 3.8] *	5.1<br>[2.7, 6.2] *	8<br>[5.2, 9.3] *
Model fit to history (probability of a residual more than 2.5% away from zero)	0.000 *	0.000 *	0.016 *
Outcome 2: Brain imaging within 45 minutes of emergency department arrival<br>Number of years (states) in the percentile	3 years<br>(n=14)	4 years<br>(n=11)	5 years<br>(n=6)
Mean difference between observed and actual outcome in percentage points [90% CI]	3.1<br>[-0.5, 6.2]	3.3<br>[-0.4, 6.3]	4.7<br>[0.4, 8.4]*
Model fit to history (probability of a residual more than 2.5% away from zero)	0.181	0.461	0.363
Outcome 3: In-hospital costs for stroke patients	3 years<br>(n=22)	7 years<br>(n=12)	9 years<br>(n=7)

	Time span after first policy		
	Short-term 25th percentile	Mid-term 50th percentile	Long-term 75th percentile
Number of years (states) in the percentile			
Mean difference between observed and actual outcome in dollars [90% CI]	-609.90 [-1,470.40, -109.60]	-1,251.70 [-1,251.70, -273.80]	-1,723.5 [1,723.5, -573.00]
Model fit to history (probability of a residual more than 2.5% away from zero)	0.055	0.226	0.401
Outcome 4: In-hospital stroke mortality rate<br>Number of years (states) in the percentile	4 years (n=19)	6 years (n=16)	11 years (n=7)
Mean difference between observed and actual outcome in deaths per 100 in-patients [90% CI]	0.2 [0, 0.4]	0.1 [-.2, 0.3]	-0.5 [-.8, -0.2]
Model fit to history (probability of a residual more than 2.5% away from zero)	0.099	0.142	0.205
Outcome 5: Age-adjusted stroke mortality<br>Number of years (states) in the percentile	5 years (n=32)	8 years (n=21)	11 years (n=10)

	Time span after first policy		
	Short-term<br>25th percentile	Mid-term<br>50th percentile	Long-term<br>75th percentile
Mean difference between observed and actual outcome in deaths per 100,000 in-patients [90% CI]	-1<br>[-2.2, 0.1]	-1.4<br>[-2.8, 0.2]	-1.6<br>[-3.3, 0]
Model fit to history (probability of a residual more than 2.5% away from zero)	0.000	0.000	0.009

[a]: Stroke System of Care; [b]: Primary Stroke Centers; *There is 90% probability that the effect is positive

Figure S1. Average estimated effect of SSOC (Stroke System of Care) policies on in-hospital stroke mortality for three percentiles of states. **A,** 25th percentile includes 19 states with 4 years of observed outcomes after policies were in effect. **B,** 50th percentile includes 16 states with 6 years of observed outcomes after policies were in effect. **C,** 75th percentile includes 7 states with 11 years of observed outcomes after policies were in effect. Year “0” on the x-axis is the year the first SSOC policy was in effect per state. Blue shaded area represents the 90% CI around the estimated effect. Pr(|res|>2.5%” indicates the posterior probability of observing a discrepancy that is larger than +/-2.5% between the predicted and observed values. These notations apply to all remaining figures.

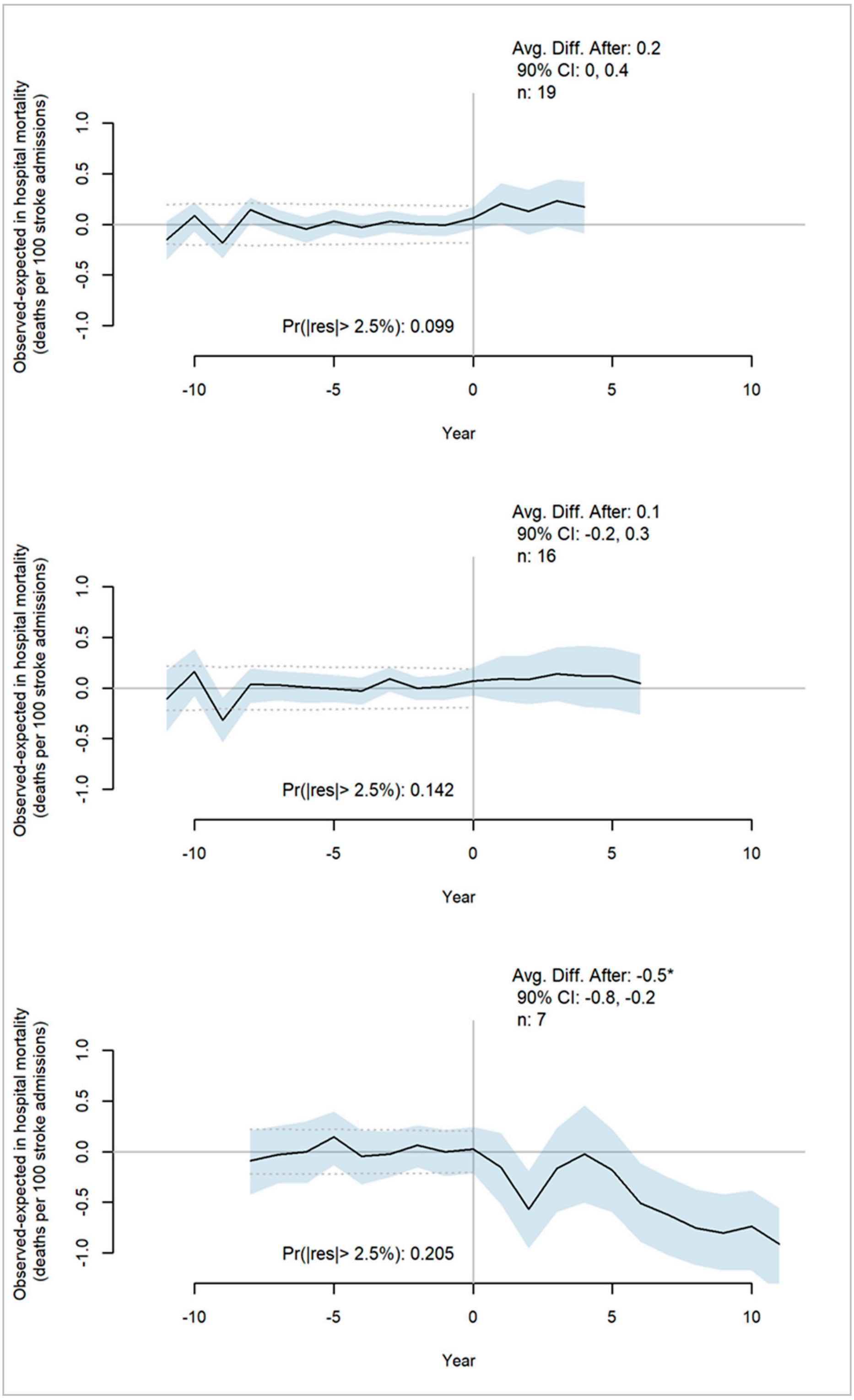

Figure S2. Average estimated effect of SSOC (Stroke System of Care**) policies on absolute difference in stroke mortality between white and non-white populations for three percentiles of states. A,** 25th percentile includes 30 states with 5 years of observed outcomes after policies were in effect. **B,** 50th percentile includes 20 states with 8 years of observed outcomes after policies were in effect. **C,** 75th percentile includes 10 states with 12 years of observed outcomes after policies were in effect.

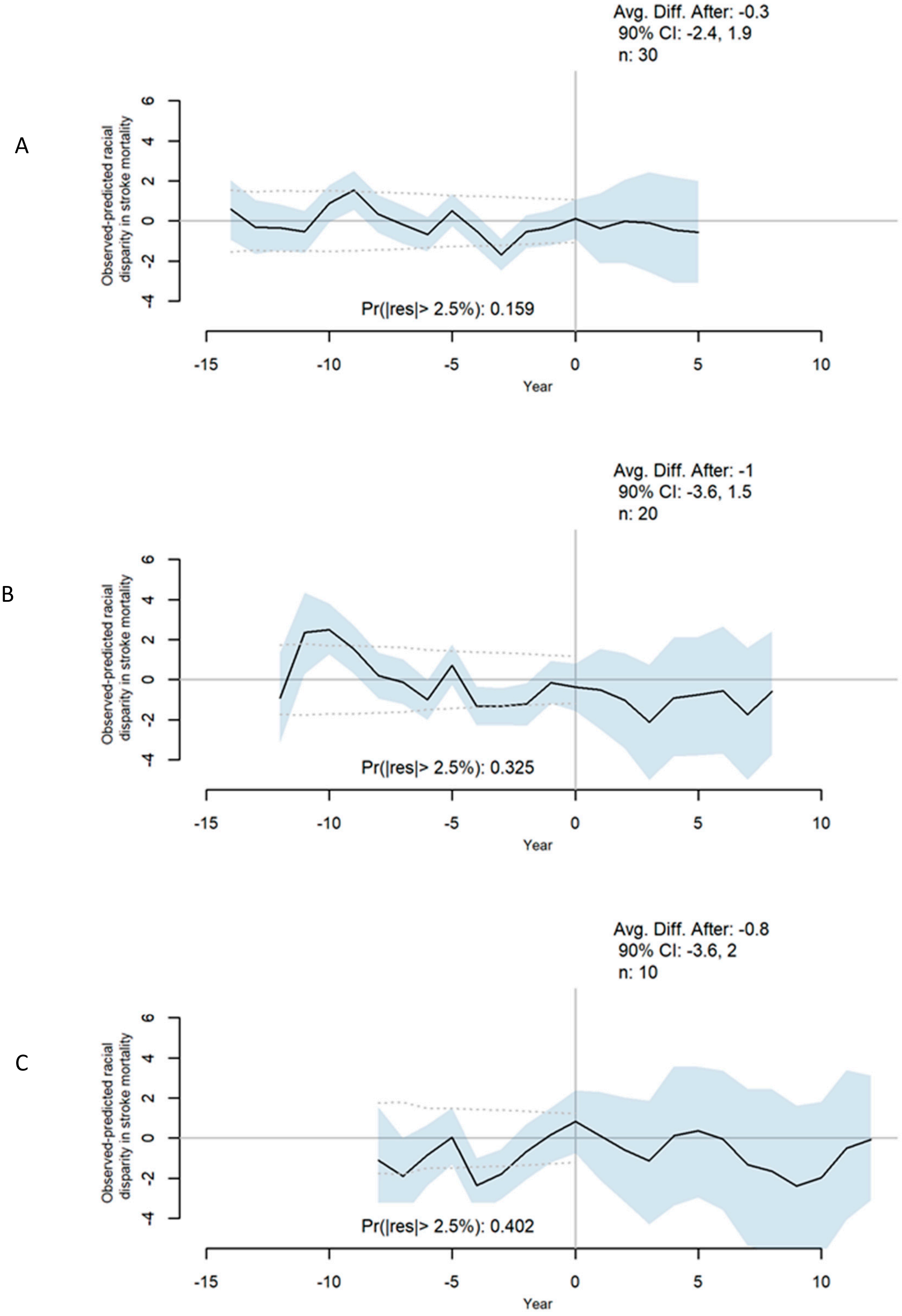

Figure S3. Average estimated effect of SSOC (Stroke System of Care) **policies on absolute difference in stroke mortality between rural and non-rural populations for three percentiles of states. A,** 25th percentile includes 29 states with 5 years of observed outcomes after policies were in effect. **B,** 50th percentile includes 18 states with 8 years of observed outcomes after policies were in effect. **C,** 75th percentile includes 13 states with 10 years of observed outcomes after policies were in effect.

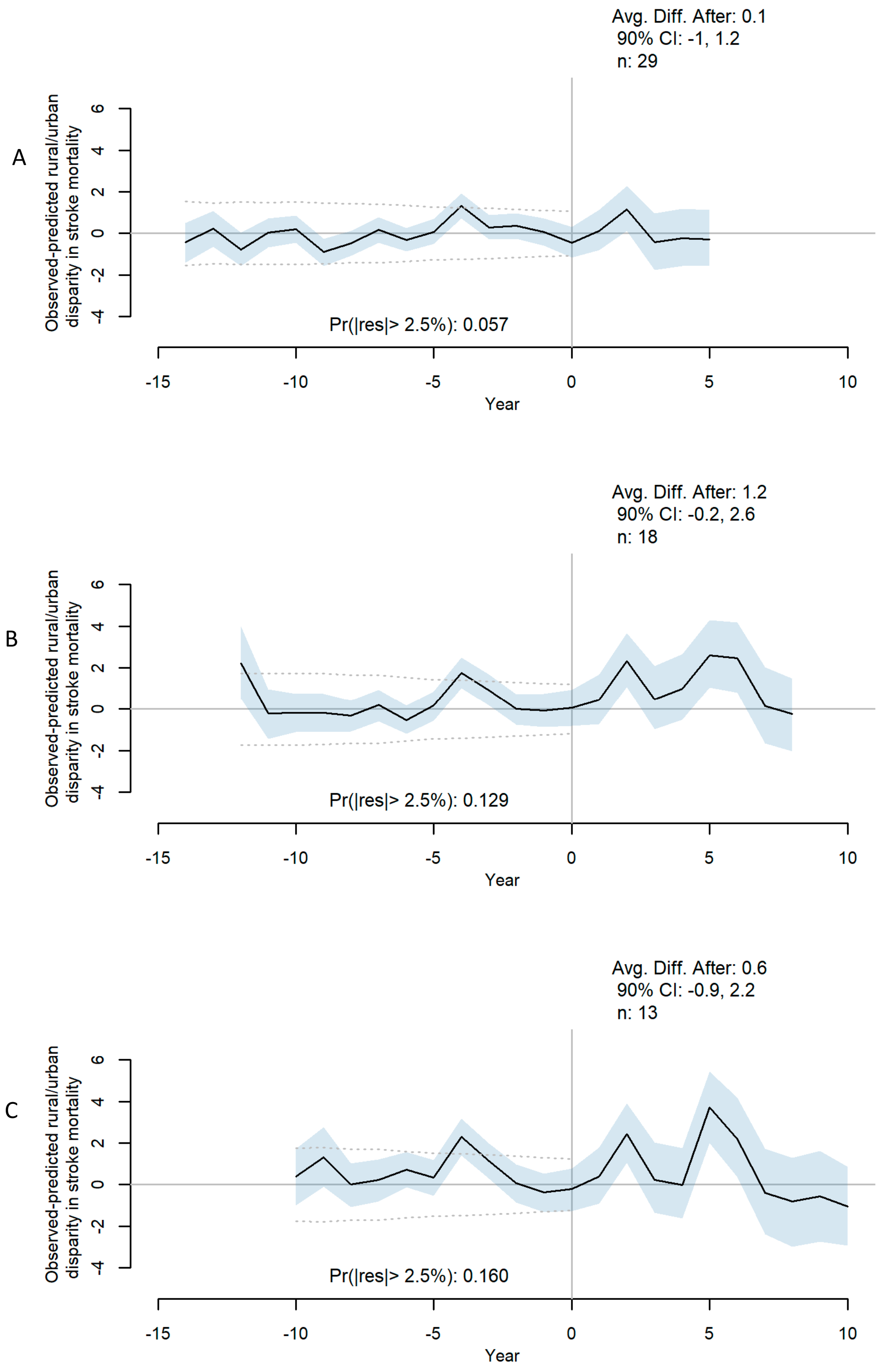

Supplement: Supplementary file 1 [file healthcare-11-02842-s001.zip › healthcare-2588860-supplementary.pdf]
